# Exploring NRB Biofilm Adhesion and Biocorrosion in Oil/Water Recovery Operations Within Pipelines

**DOI:** 10.3390/bioengineering11101046

**Published:** 2024-10-20

**Authors:** Hadjer Didouh, Hifsa Khurshid, Mohammed Hadj Meliani, Rami K. Suleiman, Saviour A. Umoren, Izzeddine Sameut Bouhaik

**Affiliations:** 1 Laboratory of Theoretical Physics and Materials Physics (LPTPM), Department of Process Engineering, Faculty of Technology, Hassiba Benbouali University of Chlef, Hay Salem 02000, Algeria; h.didouh@univ-chlef.dz (H.D.); i.sameutbouhaik@univ-chlef.dz (I.S.B.); 2 Interdisciplinary Research Center for Advanced Materials, King Fahd University of Petroleum & Minerals (KFUPM), Dhahran 31261, Saudi Arabia; ramismob@kfupm.edu.sa (R.K.S.); umoren@kfupm.edu.sa (S.A.U.); 3 Laboratory of Theoretical Physics and Materials Physics (LPTPM), Department of Mechanical Engineering, Faculty of Technology, Hassiba Benbouali University of Chlef, Hay Salem 02000, Algeria; m.hadjmeliani@univ-chlef.dz; 4 Department of Materials Science and Engineering, King Fahd University of Petroleum & Minerals (KFUPM), Dhahran 31261, Saudi Arabia

**Keywords:** corrosion, crude oil, injection water, API-5L X52, biofilm

## Abstract

Microbially influenced corrosion represents a critical challenge to the integrity and durability of carbon steel infrastructure, particularly in environments conducive to biofilm formation by nitrate-reducing bacteria (NRB). This study investigated the impact of NRB biofilms on biocorrosion processes within oil/water recovery operations in Algerian pipelines. A comprehensive suite of experimental and analytical techniques, including microbial analysis, gravimetric methods, and surface characterization, were employed to elucidate the mechanisms of microbially influenced corrosion (MIC). Weight loss measurements revealed that carbon steel samples exposed to injection water exhibited a corrosion rate of 0.0125 mm/year, significantly higher than the 0.0042 mm/year observed in crude oil environments. The microbial analysis demonstrated that injection water harbored an average of (4.4 ± 0.56) × 10^6^ cells/cm^2^ for sessile cells and (3.1 ± 0.25) × 10^5^ CFU/mL for planktonic cells, in stark contrast to crude oil, which contained only (2.4 ± 0.34) × 10^3^ cells/cm^2^ for sessile cells and (4.5 ± 0.12) × 10^2^ CFU/mL for planktonic cells, thereby highlighting the predominant role of injection water in facilitating biofilm formation. Contact angle measurements of injection water on carbon showed 45° ± 2°, compared to 85° ± 4° for crude oil, suggesting an increased hydrophilicity associated with enhanced biofilm adhesion. Scanning electron microscopy further confirmed the presence of thick biofilm clusters and corrosion pits on carbon steel exposed to injection water, while minimal biofilm and corrosion were observed in the crude oil samples.

## 1. Introduction

Algeria, the largest OPEC member country in Africa by geographic area, relies heavily on fossil energy sources to drive its economy. The nation boasts proven crude oil reserves of 12.2 billion barrels, with a production rate of 1157.1 thousand barrels per day and exports amounting to 642.2 thousand barrels per day [1,2,3].

Algerian crude oil is renowned for its high quality, characterized by low sulfur and mineral content. Sonatrach, the national oil and gas company, plays a pivotal role in the industry [3]. However, the high water content or emulsions in crude oil pose significant challenges. Water presence in crude oil hampers production, transportation, and processing, leading to increased downtime in petroleum operations. Specific issues include reduced efficiency of processing and transport vessels, corrosion of production infrastructure, and the formation of emulsions that hinder oil recovery and raise environmental concerns due to the oil content in effluent water [4,5]. Emulsions, colloidal dispersions of incompletely miscible liquids, often form stable water-in-oil mixtures due to agitation during production [5,6,7]. Water is essential for oil extraction and refinery processes, yet it can exacerbate corrosion and other problems. In the industry, standards for pipelines, such as BS&W values and salt content, must be rigorously maintained [8].

Natural crude oil reservoirs contain multiphase fluids, including crude oil, gas, and produced water. These reservoirs encompass diverse ecological niches inhabited by various thermophilic and mesophilic archaea and bacteria that can metabolize organic and inorganic compounds within the crude oil and metal pipelines under extreme environmental conditions [9,10]. Such microbial communities have the potential to alter the permeability and chemistry of geological systems in the oil reservoir environment [11,12]. Produced water discharged from oil reservoirs is rich in minerals, primarily chloride and sulfate, which modify microbial metabolism and initiate corrosion processes. Biofilm formation on metal surfaces significantly accelerates this corrosion [11,12,13].

Produced water represents the most significant waste stream generated by the oil and gas industry throughout the production, recovery, and transportation phases of crude oil extraction [14]. While the composition of produced water can vary markedly from one oilfield to another, it predominantly consists of a complex mixture of dissolved organic and inorganic compounds, dispersed hydrocarbons, dissolved gases, various chemicals, suspended solids, and microorganisms [15]. This intricate chemical milieu fosters the growth of diverse microbial populations [16]. Notably, a variety of microorganisms including iron precipitating bacteria, sulfate-reducing bacteria (SRB), sulfur-oxidizing bacteria, exopolysaccharide (EPS) producers, fermentative organisms, methanogens, as well as species from the genera Pseudomonas, Bacillus, Archaea, and fungi can be isolated from produced water associated with oil extraction [17,18].

Specific genera of sulfate-reducing bacteria, including Desulfomicrobium, Desulfovibrio, Desulfohalobium, Desulfococcus, Desulfosarcina, Desulfobacter, Desulfobacterium, and Desulfobulbus, along with iron-reducing bacteria such as Desulfuromusa, Pelobacter, Malonomonas, and Desulfuromonas, have been identified in produced water samples [18]. The coexistence of these microbial species in a mixed consortium can initiate corrosive processes, as the interaction among microbial cells catalyzes biofilm formation on metal surfaces [19]. The accumulation of macromolecules within the produced water, combined with the transport of planktonic cells to metallic surfaces, triggers the onset of biofilm development [20]. Within this microenvironment, microorganisms release toxic metabolites, generate EPS, produce corrosive acids (both organic and inorganic), and volatile compounds such as ammonia and hydrogen sulfide, thereby disrupting the corrosion cell through the utilization of hydrogen, oxygen, or iron [21,22,23,24].

Oil and gas pipelines, as well as storage tanks, are typically constructed from carbon and low-alloy steels known for their high mechanical strength, yet these materials are susceptible to both internal and external corrosion [25,26,27,28]. It is estimated that 20% of economic losses in oil and gas production are due to microbial corrosion [29]. Microbially influenced corrosion (MIC), or biocorrosion, results from the activities of microorganisms. The interactions between the microorganisms within the biofilm and the metal surface, driven by weight loss, lead to metal oxidation [30,31,32]. MIC is commonly observed in internal pipelines, where microbial metabolites facilitate electron transfer through the oxidation of elemental iron on the metal surface [33,34,35].

Biofilms are complex, biologically active matrices composed of cells and extracellular substances associated with solid surfaces [36]. These sessile microbial communities thrive on surfaces and are often embedded in a matrix of extracellular polymeric substances (EPSs) [37]. A biofilm can be defined as a microbially derived sessile community characterized by cells that attach to an interface and are embedded in an exopolysaccharide matrix, exhibiting an altered phenotype [38]. Within these biofilms, microcolonies are discrete, matrix-enclosed communities of bacterial cells, which can consist of one or multiple species. Depending on the species involved, the microcolony composition typically ranges from 10 to 25% cells and 75 to 90% EPS matrix [39]. The bacterial cells in the matrix are noted for their lack of Brownian motion, and detailed structural analysis of many microcolonies frequently reveals a mushroom-like shape [40].

The corrosion processes in oil/water systems are complex and multifaceted. Key mechanisms include electrochemical corrosion, MIC, CO_2_ corrosion, and H_2_S corrosion. The presence of water creates an electrolyte, facilitating electrochemical reactions at the metal surface of pipes and equipment. Injection water introduces or supports the growth of microorganisms that contribute to corrosion through their metabolic activities. The economic impact of corrosion in oil production is substantial, encompassing increased maintenance and replacement costs, production downtime for repairs and inspections, and potential environmental and safety incidents due to equipment failure.

Punniyakotti Elumalai et al. examined the microbial biofilm community causing corrosion in API 5L carbon steel exposed to produced water from Indian oil reservoirs. 16S rRNA sequencing revealed that Proteobacteria, particularly *Marinobacter hydrocarbonoclasticus* and *Marinobacter alkaliphilus*, dominated the biofilm. Surface analysis confirmed dense biofilm formation and severe pitting, while X-ray diffraction identified increased corrosion products, highlighting the critical role of microbial biofilms in accelerating carbon steel corrosion in oil/water environments [41].

Edkarlla S. D. de Oliveira et al. investigated the role of biofilm-forming microorganisms in the corrosion of X80 steel exposed to produced water for 360 days. The sessile cell count was measured at 7.39 × 10⁴ CFU/cm^2^, and the SEM results showed salt crystals, exopolysaccharides, and corrosion products. Weight loss analysis indicated severe corrosion in biotic systems (0.3039 g/cm^2^) compared to abiotic systems (0.0222 g/cm^2^) [42].

Research has indicated that fluid velocity within pipelines significantly influences biofilm formation. Specifically, lower flow rates facilitate biofilm development, increasing the susceptibility of steel to MIC and pitting corrosion, whereas higher flow rates promote the formation of a protective layer of corrosion products on steel surfaces [43]. Jin and Guan [44] studied the impact of iron ions on biofilm formation and EPS production, revealing that moderate concentrations of iron facilitate both biofilm development and EPS production.

Cote et al. observed a reduction in bacterial diversity following exposure to pigging debris in a mixture of artificial seawater and produced water, suggesting that corrosion in steel is not exclusively driven by SRB and Clostridiales [45]. Additionally, Liu et al. identified the nitrate-reducing bacterium Brevibacterium frigoritolerans as a contributor to the deepening of pits in X80 steel, destabilizing the protective layer of corrosion products and functioning as a biological cathode within the biofilm by reducing nitrate [46].

This study focuses on the Hassi Messaoud oil field in southern Algeria. The Cambrian sandstone formation, located at a depth of 11,000 feet, has a pay zone between 100 and 300 feet thick and covers approximately 600 square miles. The original reservoir pressure was 6825 psi, with the crude bubble point fluctuating between 2880 psi and 2130 psi across the field.

Water-flooding, a commonly used secondary recovery method in the Hassi Messaoud field, aims to maintain reservoir pressure and enhance oil recovery. The effectiveness of this technique depends on several key parameters, including the geometry and geology of the reservoir, petrophysical properties (porosity and permeability), fluid properties (viscosity and wettability), and mineralogical properties (clays in the reservoir rock) [47].

The MIC represents a critical challenge to the integrity and operational efficiency of carbon steel pipelines, particularly in the oil and gas sector. While the interactions between crude oil and injection water are known to significantly influence biofilm development, there remains a limited understanding of the specific factors facilitating this process. Past studies have predominantly focused on general corrosion mechanisms, often overlooking the critical role that nitrate-reducing bacteria (NRB) biofilms play in the corrosion of pipelines, especially in environments such as the Hassi Messaoud oilfields in Algeria, where unique conditions prevail.

Despite the growing recognition of NRB biofilms as key contributors to MIC, limited research has quantitatively examined their specific impact within oil/water recovery operations. Additionally, the complex physicochemical interactions between Algerian crude oil and injection water, crucial for understanding MIC dynamics, have not been thoroughly explored. This knowledge gap hinders the development of effective strategies for detecting, monitoring, and mitigating biocorrosion in pipelines.

This study seeks to address this gap by employing a comprehensive approach that combines microbial analysis, gravimetric methods, and advanced surface characterization techniques, such as scanning electron microscopy (SEM), to evaluate the impact of microbial diversity within biofilms on the corrosion behavior of API 5L X52 carbon steel in oilfield environments. By immersing carbon steel coupons in crude oil and produced water from Algerian reservoirs under controlled laboratory conditions, this research aims to systematically investigate the roles of crude oil and injection water in promoting NRB biofilm adhesion and the subsequent biocorrosion of carbon steel. The findings are expected to provide valuable insights into the mechanisms of MIC and contribute to the development of more effective corrosion mitigation strategies, thereby improving the safety and longevity of oil transportation pipelines.

## 2. Materials and Methods

In this study, a comprehensive approach was undertaken to assess biofilm adhesion and corrosion on carbon steel in crude oil and injection water environments. The experimental procedures included sample preparation, bacterial culture, biofilm formation assessment, and surface analysis.

### 2.1. Sample Preparation

#### 2.1.1. Crude Oil/Injection Water

Hassi Messaoud (HMD) field is situated in southeast Algeria and was discovered in 1956 with its reserves (OOIP) of several billion m^3^ in place. The productive formation is a Cambrian sandstone, 3400 m deep with an average thickness of 300 m and three productive layers (R2, Ra, and Ri from bottom to top). HMD is mainly produced by solution gas drive [48]. Algerian crude oil is renowned for its high quality, characterized by low sulfur and mineral content.

**Figure 1:**
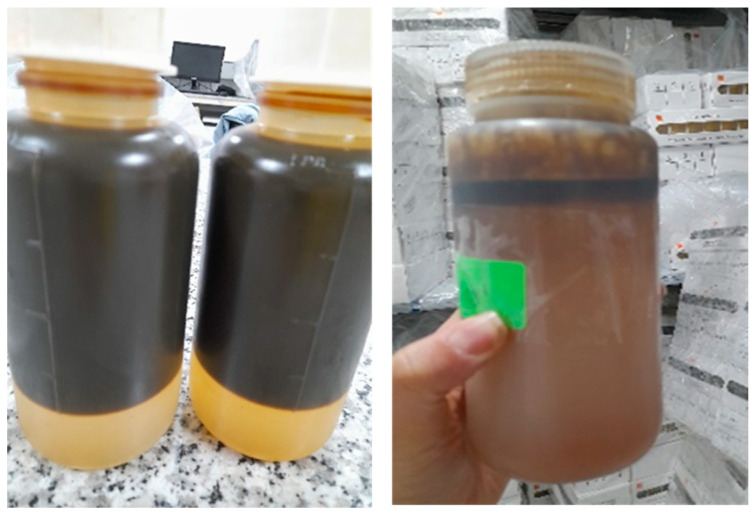
Algerian crude oil/injection water.

The injection water sample utilized in this study was obtained from the oilfield of Hassi Messaoud in southern Algeria. The sample (Figure 1) was collected under aseptic conditions at 45 °C and a pH of 6.9. The collected oil/water samples were transported to the laboratory in sterile glass flasks, rinsed three times with the sampled water, and stored at 4 °C. The sample was not treated with any chemicals. Upon arrival, the samples underwent phase separation, with the oil and water layers being isolated for further processing. The samples were subjected to physicochemical analyses.

#### 2.1.2. Carbon Steel Preparation

API 5L X52 specimens were prepared with dimensions of 1 × 1 × 1 cm^3^ and coated with epoxy, leaving only one surface exposed. These samples underwent mechanical polishing to achieve a 1200-grit surface finish. Following polishing, the samples were thoroughly cleaned with acetone to ensure a uniform and homogeneous surface. API 5L X52 grade steel is widely utilized in piping applications within the oil and gas industry due to its favorable mechanical properties and resistance to corrosion. The chemical composition of X52 steel is as follows: Carbon (0.22%), Manganese (1.22%), Silicon (0.24%), Chromium (0.16%), Nickel (0.14%), Molybdenum (0.06%), Sulfur (0.04%), Copper (0.19%), Titanium (0.04%), Niobium (<0.05%), and Aluminum (0.32%), with the balance being Iron (Fe).

### 2.2. Bacterial Strain Culture and Condition

#### 2.2.1. NRB Bacteria

Nitrate-reducing bacteria (NRB) were cultured using the Coleville Synthetic Brine (CSB) medium, tailored to match the properties of injection water and the specific growth requirements of NRB [49].

#### 2.2.2. Bacterial Cultures

**Figure 2:**
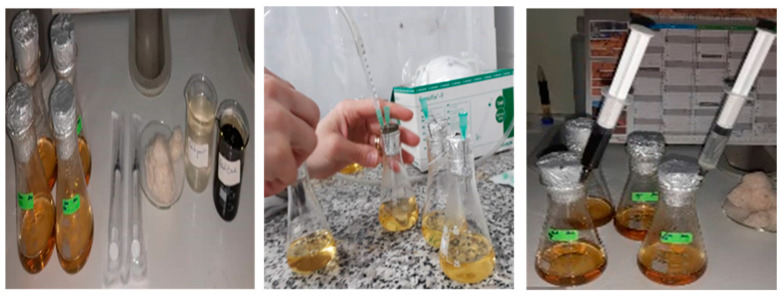
Experimental setup.

For the preparation of Luria–Bertani (LB) broth as the growth medium, a mixture of 10 g of peptone, 5 g of yeast extract, and 10 g of NaCl was made, and 48 Erlenmeyer flasks were set up, with half of these designated for anaerobic conditions through nitrogen purging and the other half left unaltered for aerobic conditions. After autoclaving for 20 min at 120 °C, the aerobic flasks were incubated at 37 °C for 3 days, followed by media refreshment to obtain a pure stain. All the flasks were inoculated with 10% formation water/crude oil containing different aerobic and anaerobic bacteria Figure 2) [50].

#### 2.2.3. CSB Media

The Coleville Synthetic Brine (CSB) medium was selected for culturing nitrate-reducing bacteria due to its compatibility with the properties of injection water and the growth requirements of these bacteria as described by Fan et al. [49]. The medium comprises various essential compounds in the following concentrations: 0.02 g/L of ammonium chloride, 0.24 g/L of calcium chloride, 0.68 g/L of magnesium sulfate, 0.027 g/L of monopotassium phosphate, 1 g/L of potassium nitrate, 7 g/L of sodium chloride, 0.68 g/L of sodium acetate, 0.0001 g/L of resazurin, and 1.90 g/L of sodium bicarbonate. Each compound was carefully weighed and dissolved in 1L of distilled water. The pH of the solution was adjusted to a range of 7.2–7.5 using a 20% HCl solution. Following thorough mixing, the medium was aliquoted into 10 mL portions in penicillin-type vials, which were subsequently capped and sterilized by autoclaving at 120 °C for 20 min at 1 bar. To ensure the anaerobic conditions necessary for NRB growth, the vials were purged with nitrogen gas for 10 s prior to autoclaving [49].

**Figure 3:**
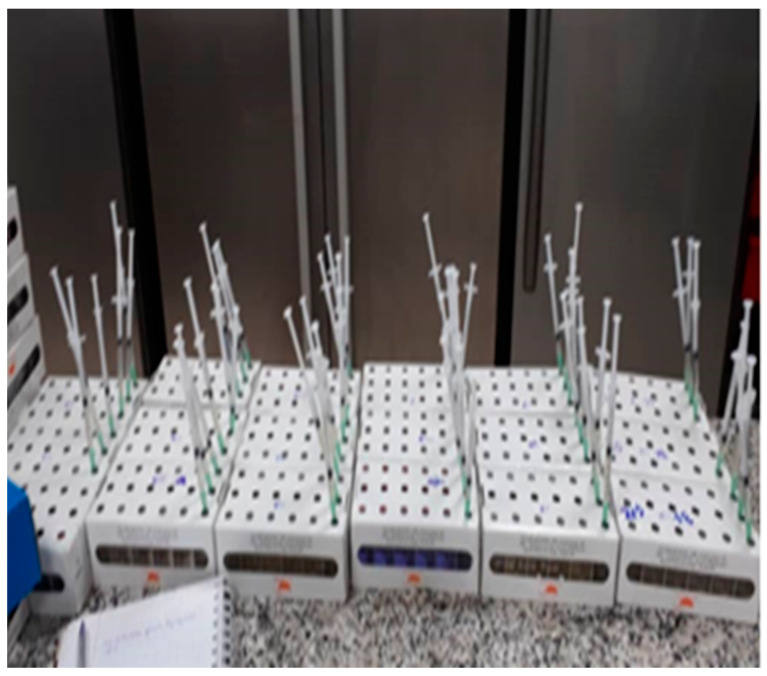
Inoculation and incubation test.

Sterile CSB medium in penicillin-type vials were inoculated with 1 mL of injection water/crude oil using a sterile syringe, creating a 10^−1^ dilution. This was repeated up to 10^−6^ dilution. The presence of NRB was indicated by microscopic observation, a pink color change, and bubble formation from nitrate reduction. Kits of six vials were incubated for 72 h at 37 °C (Figure 3).

### 2.3. Adhesion Essay

Following the preparation and regeneration processes, carbon steel coupons were introduced into separate bottles containing injection water and crude oil. These setups were then incubated at 37 °C for 7 days to investigate biofilm formation and adhesion. Throughout the incubation, both sessile and planktonic microbial populations were monitored to assess the extent of biofilm development. After the incubation period, the coupons were carefully extracted and subjected to a series of analyses, including scanning electron microscopy (SEM) measurements, to quantitatively and qualitatively evaluate the biofilm’s adhesion on the carbon steel surfaces.

#### 2.3.1. Sessile and Planktonic Cells

Biofilm formation on carbon steel surfaces in oilfield environments is a significant cause of biocorrosion, leading to material degradation and operational inefficiencies [21,51,52]. Biofilms, composed of sessile microbial cells embedded in a matrix of extracellular polymeric substances (EPSs), adhere to surfaces and create microenvironments that facilitate corrosion processes [53]. Planktonic cells, or free-floating microorganisms, also play a role by replenishing the biofilm community and contributing to the corrosion process through metabolic activities. Bacterial cell quantification was conducted at 7 days of exposure to the biotic system. The colony-forming unit (CFU) method was used to quantify the planktonic cells within the microbial community.

#### 2.3.2. Surface Analysis

**Figure 4:**
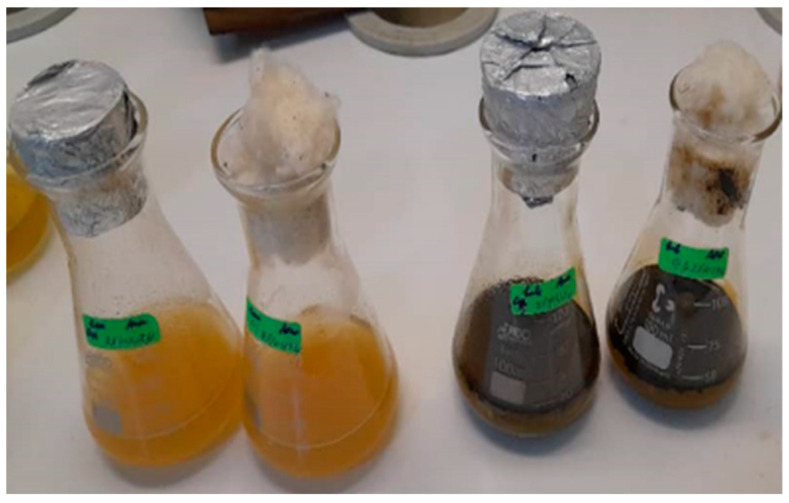
Preparation of specimens for the surface analysis.

After the incubation period (Figure 4), the carbon steel coupons were carefully removed from the flask and subjected to surface analysis using scanning electron microscopy and energy-dispersive X-ray spectroscopy (SEM/EDX) with the QUANTA 650 model to observe and characterize the biofilm formation and corrosion features at a microscale level.

### 2.4. Weight Loss

Weight loss measurements were employed to quantitatively evaluate the corrosion rates of carbon steel specimens in crude oil and injection water environments. Four replicate carbon steel coupons were weighed and then immersed in each fluid environment for 28 days at 37 °C, using a thermostatically controlled water bath. To minimize dissolved oxygen levels, both fluids were deoxygenated by bubbling high-purity nitrogen gas through them for 10 min prior to immersion. Post-exposure, the coupons were cleaned with Clarke’s solution as per ASTM G1-03 protocol to remove corrosion products and then weighed to determine weight changes.

### 2.5. Contact Angle

The contact angle measurements in the presence of crude oil and injection water, stainless steel, and carbon steel samples were prepared and analyzed using a contact angle goniometer. The stainless steel and carbon steel samples were cleaned and dried using standard procedures, before being polished and rinsed in an ultrasonic cleaner and dry ethanol. The crude oil and injection water samples were prepared as received. In the experimental setup, a 5 µL droplet of crude oil was carefully deposited onto the surface of each material, and the contact angle was measured using the goniometer. The same procedure was repeated with the injection water to assess the impact of different fluids on biofilm adhesion.

## 3. Results and Discussion

### 3.1. Physicochemical Analysis of Injection Water

Injection water is considered a favorable environment for various microbial populations due to its richness in essential growth elements. A physicochemical analysis is performed to determine its composition. Hassi Messaoud water has a yellowish color and no odor. The chemical analysis results are shown in Table 1.

In accordance with the guidelines established by the international Organization of normalization (ISO), the physicochemical analysis of formation water was conducted following the normative references outlined in the relevant standards. The pH was measured using ISO 10523 [54], while calcium ions (Ca^2+^) and magnesium ions (Mg^2+^) were quantified using ISO 6058-1984(E) and ISO 6059-1984(E) [55], respectively. Chloride ions (Cl^−^) were analyzed according to NF ISO 9297-2000 [56].Potassium ions (K^+^) and sodium (Na^+^) concentrations were determined following NFT 90-019 [57]. Dry extract analyzed using Water analysis, J. Rodier Dunod 1976 P 35. Sulphate ions (SO_4_^2−^) were analyzed using Spectrophotometry method.

Water has a neutral pH, favoring NRB multiplication. The presence of nitrate and nitrite ions indicates a nitrogen cycle and NRB presence. The lower dissolved oxygen content supports the denitrifying activity. Salinity, mineral elements, and trace elements are adequate for NRB growth, while sulfates and CO₂ stimulate SO-NRB [58].

The physicochemical analysis of the injection water from Hassi Messaoud (HMD) reveals various concentrations of essential chemical elements that influence water quality and its potential impact on corrosion processes and biofilm formation in pipelines. For instance, the high concentration of chloride is concerning because chloride ions are well known for their ability to accelerate corrosion processes, especially in environments containing iron and steel [59]. Additionally, the presence of sulfates can also promote corrosion by forming sulfuric acids under anaerobic conditions, as supported by the findings of [60]. Calcium ions and magnesium ions contribute to water hardness, potentially leading to the formation of scale deposits on pipeline surfaces. The presence of nitrates and nitrites indicates microbial activity, particularly of nitrifying and denitrifying bacteria, which can influence corrosion.

Igunnu and Chen report that the solids found include corrosion products, precipitated solids, carbonates, and other particulates [61]. The accumulation of these solids can lead to serious operational issues, such as the formation of emulsions and clogging of pipelines. Additionally, the content of oil and grease in the water decreased, likely due to the microbiological biodegradation of hydrocarbons. Several studies have shown that biodegradation is an effective biological treatment method for the removal of hydrocarbons from injection water. However, its efficiency diminishes in highly saline conditions [62]. These results indicate that injection water is a dynamic medium with a complex chemical composition that varies over time due to environmental factors such as temperature, air exposure, photo-oxidation, biodegradation, and other system-specific conditions [15]. The literature also suggests that seasonal variations may influence the physicochemical parameters of injection water in oilfields [63].

### 3.2. Reactivation of NRB Strains in CSB Liquid Medium

**Figure 5:**
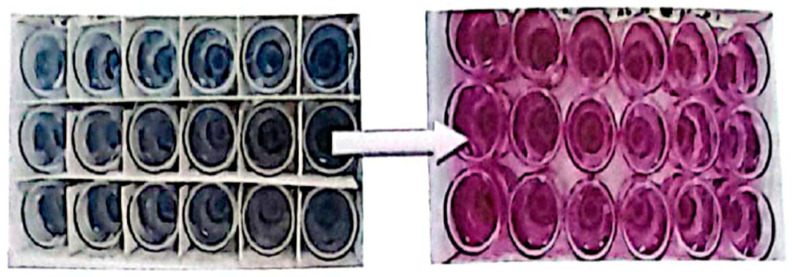
Test kit for NRB bacteria.

The presence of NRB in the CSB medium was indicated by a pink color change shown in Figure 5, signifying nitrate reduction. After incubation at 37 °C, the contamination rates of water samples were assessed by measuring NRB concentrations. The results demonstrated significant NRB contamination in the injection water samples, whereas no contamination was observed in the crude oil samples. These presented in Table 2 indicate that the injection water is primarily responsible for NRB presence and, consequently, the associated corrosion issues. Nitrite assays conducted (CSB) media demonstrate a significant increase in the abundance of sessile cells under anaerobic conditions associated with injection water. Nitrate reductase presence is confirmed by pink/purple coloration with reagents, indicating nitrate reduction capability.

Notably, nitrite assays conducted (CSB) media demonstrate a significant increase in the abundance of sessile cells under anaerobic conditions associated with injection water.

### 3.3. Adhesion Essay

#### 3.3.1. Sessile and Planktonic Cell

A significant disparity was observed in microbial colonization between injection water and crude oil. X52 coupons incubated in injection water exhibited a sessile cell count of (4.4 ± 0.56) × 10^6^ cells/cm^2^. In contrast, coupons immersed in crude oil showed a markedly lower sessile cell count of (2.4 ± 0.34) × 10^3^ cells/cm^2^. This difference of three orders of magnitude suggests that the injection water environment is substantially more conducive to bacterial attachment and potential biofilm formation [64]. Similarly, planktonic cell concentrations differed significantly between the two environments. In injection water, the planktonic cell count was (3.1 ± 0.25) × 10^5^ CFU/mL, while in crude oil, it was (4.5 ± 0.12) × 10^2^ CFU/mL. This disparity of approximately three orders of magnitude aligns with the trend observed in sessile cell counts [65].

#### 3.3.2. Surface Analysis

**Figure 6:**
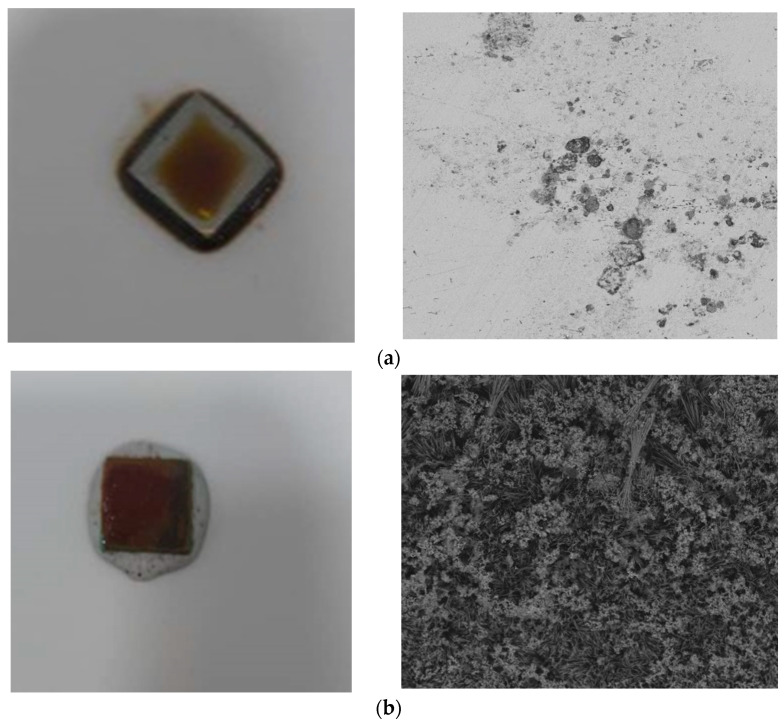
Top-surface SEM images for X52 carbon steel in the presence of (**a**) crude oil and (**b**) injection water.

Scanning electron microscopy (SEM) was employed to examine biofilm formation on the metal surfaces. The SEM analysis of NRB biofilms on carbon steel showed heterogeneity among bacterial types and the presence of EPS. Biofilm formation modifies the metal–solution interface, creating micro-niches with different physicochemical conditions, affecting corrosion [66]. The images of steel exposed to crude oil (Figure 6a) reveal relatively smooth surfaces with minimal microbial presence, characterized by occasional small particles and minor irregularities; crude oil displayed minimal biofilm formation, indicating the presence of a stable passivation layer on the metal surface, attributable to the low chloride content. In stark contrast, the SEM images of steel exposed to injection water (Figure 6b) confirmed the presence of a thick biofilm in injection water. Cells aggregated and expanded as clusters associated with imperfections on the carbon steel surfaces, forming asymmetric clumps scattered over the surface. Intermittent stretched-branch, ball, and net-like structures of aggregated cells were observed, clearly indicating microbial attachment on the coupon surfaces. These images showcase irregular surface topography with numerous protrusions and depressions, clustered formations resembling microbial cells or extracellular polymeric substances, and potential corrosion products or mineral deposits interspersed within the biological structures. The marked difference between these two sets of images corroborates the quantitative data previously discussed, which indicated significantly higher sessile and planktonic cell counts in the injection water environment [21,22,67]. Biofilm development is a highly dynamic process, heavily influenced by hydrodynamic conditions. The interaction of microorganisms with the substrate, biofilm thickness development, and its eventual detachment are all stages of biofilm formation within pipelines, where flow plays a critical role. The velocity of the flow inside pipelines impacts mass transfer and shear stress on the surface, which can either facilitate or hinder biofilm adhesion to the substrate [42].

### 3.4. Weight Loss

In this study, a weight loss experiment was conducted to simulate the biocorrosion reaction, with continuous monitoring throughout the process. As the reaction progressed, the injection water gradually turned light yellow. After two days of immersion, a visible, dense biofilm was noticed on the carbon steel surface, and the injection water became turbid due to the increase in free-floating microorganisms. The biofilm’s thickness increased with the incubation period. By the end of the weight loss experiments, a dense biofilm was observed, as shown in Figure 6b.

The corrosion of X52 in Algerian crude oil and injection water at 37 °C is significantly influenced by microbial activity, with nitrate-reducing bacteria (NRB) playing a crucial role along with other microorganisms. NRB are of particular interest due to their ability to use nitrate as an alternative electron acceptor in anaerobic conditions, which are common in oil and gas environments [68]. The corrosive impact of NRB varies significantly between sessile (biofilm-associated) and planktonic (free-floating) populations. The corrosion rates were calculated to be 0.0125 mm/year for coupons incubated with injection water and 0.0042 mm/year for coupons incubated with crude oil, respectively [69]. This increased corrosion rate with injection water in the biotic systems can be attributed to the hyper-salinity of the injection water, which likely facilitated enhanced corrosion. The enhanced corrosivity of sessile NRB can be attributed to several factors. Within biofilms, NRB creates microenvironments with chemical gradients, including nitrate and nitrite concentrations, pH, and redox potential, which accelerate localized corrosion. The extracellular polymeric substances (EPSs) produced by sessile NRB concentrate corrosive ions and metabolites at the metal–biofilm interface, further exacerbating corrosion [21]. Moreover, recent studies have shown that some NRB directly utilize elemental iron as an electron donor, a process known as electrical microbially influenced corrosion (EMIC), which is more pronounced in sessile communities.

### 3.5. Contact Angle

Contact angle analysis was performed to evaluate the wettability of the steel surfaces, indicating the extent of hydrophobic or hydrophilic interactions influenced by biofilm formation. The results revealed distinct differences in biofilm formation, contact angles, and corrosion rates between steel surfaces exposed to injection water and those immersed in crude oil.

Coupons exposed to injection water exhibited significantly higher biofilm formation, lower contact angles, and higher corrosion rates compared to those immersed in crude oil. The injection water samples displayed dense biofilm clusters and pronounced corrosion pits, indicative of substantial microbial activity and associated corrosion processes. In contrast, the crude oil samples showed minimal biofilm presence and corrosion. The contact angle measurements further highlighted the differences between the materials.

#### 3.5.1. Injection Water and NRB Interaction

**Figure 7:**
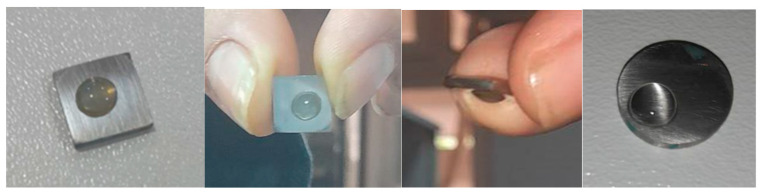
Contact angle images of the tested coupons.

X52 Carbon Steel (API 5L X52):

The contact angle of injection water on X52 carbon steel in the presence of NRB dropped from an initial 72° ± 3° to 45° ± 2°. This reduction in contact angle indicates a transition toward hydrophilicity, driven by biofilm formation and microbial adhesion facilitated by NRB. The corrosion rate for X52 carbon steel in this environment was measured at 0.068 mm/year, reflecting significant microbial activity contributing to biocorrosion. Dense biofilm clusters were observed (Figure 7), particularly at the 6, 9, and 12 o’clock positions, where contact angle values were slightly lower at 43° ± 2° due to gravity’s influence on water adhesion.

Stainless Steel (316L SS):

In contrast, stainless steel showed more resistance to biofilm adhesion and corrosion. The contact angle in injection water was recorded at 67° ± 2°, only marginally reduced from the initial value of 70° ± 3°. This suggests limited hydrophilic interaction and reduced biofilm formation compared to carbon steel. The corresponding corrosion rate was significantly lower, at 0.015 mm/year, indicating that stainless steel is much less susceptible to NRB-driven biocorrosion in injection water.

#### 3.5.2. Algerian Crude Oil and NRB Interaction

X52 Carbon Steel (API 5L X52):

When exposed to Algerian crude oil in the presence of NRB, the contact angle on X52 carbon steel remained relatively high at 85° ± 4°, indicating poor wetting and minimal hydrophilic interactions. This resulted in minimal biofilm formation and a notably lower corrosion rate of 0.008 mm/year. The inability of crude oil to adhere to the carbon steel surface prevented the development of a stable biofilm, reducing the corrosion potential.

316L Stainless Steel (316L SS):

Similar to carbon steel, stainless steel exhibited poor interaction with Algerian crude oil, with a contact angle of 82° ± 3°. This high contact angle suggests that crude oil does not readily adhere to the stainless-steel surface, preventing biofilm formation. The corrosion rate in this environment was negligible, at 0.002 mm/year, further emphasizing the superior corrosion resistance of stainless steel in crude oil environments.

## 4. Conclusions

This study highlights the delicate balance between enhanced oil recovery (EOR) techniques and the growing challenges of corrosion in aging oilfields. While EOR strategies are crucial for maintaining production levels, the findings from this research reveal a significant threat to pipeline integrity posed by nitrate-reducing bacteria (NRB) in injection water. Detailed analyses of NRB growth kinetics, biochemical properties, and biofilm formation on carbon steel in Algerian oilfields underscore the complex microbial interactions that accelerate corrosion. Weight loss measurements demonstrate a substantial difference in corrosion rates, with carbon steel surfaces exposed to injection water experiencing a corrosion rate of 0.0125 mm/year, markedly higher than the 0.0042 mm/year observed in crude oil environments. This indicates the heightened susceptibility of X52 carbon steel to biofilm formation and biocorrosion, especially in injection water where NRB activity is prominent. Conversely, 316L stainless steel exhibits a much higher resistance to biofilm adhesion and corrosion in the same environment, even in the presence of NRB. The pronounced disparity between corrosion rates in injection water and crude oil environments underscores the aggressive nature of NRB biofilms in promoting corrosion. This finding highlights the critical need for advanced, targeted corrosion management strategies, particularly for carbon steel in EOR operations. The ability of NRB communities to thrive and form biofilms on carbon steel calls for renewed efforts in developing specialized corrosion inhibitors and biocides tailored to mitigate biofilm-induced corrosion. Future research should focus on optimizing these interventions to enhance the longevity and safety of pipeline infrastructure in oilfields.

## Figures and Tables

**Table 1:** Physicochemical analysis of injection water.

Parameters	Operating Procedures	mg/L
pH	Measurement of pH by electrometry	7.1
Ca^2+^	Calcium determination by complexometry	210
Mg^2+^	Magnesium determination by complexometry	70
SO_4_^2−^	Sulphate determination	600
Cl^−^	Chloride determination by volumetry	420
Dry extract	Dry residue determination at 105 °C	165,442
K^+^	Potassium determination	40
Na^+^	Sodium determination	250
HCO_3_^−^		170
NO_3_^−^		3.23
NO_2_^−^		1.058

**Table 2:** NRB growth in HMD crude oil/injection water over 7 days in test kit.

Reading Time	Sample	Aerobic (CFU/mL)	Anaerobic (CFU/mL)
Day 1	Crude oil	10^1^	10^0^
	Injection water	10^2^	10^1^
Day 2	Crude oil	10^2^	10^1^
	Injection water	10^3^	10^2^
Day 3	Crude oil	10^2^	10^2^
	Injection water	10^3^	10^3^
Day 4	Crude oil	10^3^	10^2^
	Injection water	10^4^	10^5^
Day 5	Crude oil	10^3^	10^3^
	Injection water	10^5^	10^5^
Day 6	Crude oil	10^3^	10^3^
	Injection water	10^6^	10^6^
Day 7	Crude oil	10^3^	10^3^
	Injection water	10^6^	10^6^

## Data Availability

The original contributions presented in this study are included in this article. Further inquiries can be directed to the corresponding author.
